# Effect of Patient Age on Platelet-Rich Plasma (PRP) and Fibrin Treatments for Skin Density and Thickness: A Single-Center Ultrasound Study

**DOI:** 10.3390/life15020308

**Published:** 2025-02-17

**Authors:** Lidia Majewska, Jacek Kijowski, Karolina Dorosz

**Affiliations:** 1 ESME Clinic, 30-548 Kraków, Poland; 2 Stem Cell Laboratory, Małopolska Centre of Biotechnology, Jagiellonian University, 30-387 Kraków, Poland; jacek.kijowski@uj.edu.pl; 3 Research Assistant, Biological Sciences Department, University of Chicago, Chicago, IL 60637, USA; kdorosz@uchicago.edu

**Keywords:** platelet-rich plasma (PRP), platelet-rich fibrin (PRF), injectable platelet-rich fibrin (I-PRF), skin aging, PRP preparation, skin density, skin thickness, ultrasound evaluation, age-related skin changes

## Abstract

Objective: This study aimed to establish a reproducible protocol for obtaining four plasma fractions (autologous platelet-rich plasma (C-PRP, PRP LCC) and platelet-rich fibrin (I-PRF, F-PRF)) from a single blood draw and to evaluate their effectiveness in enhancing skin density and thickness in facial aesthetic treatments across different age groups. Methodology: Twenty participants aged 30–60 years received three treatments at 4–6 week intervals, with C-PRP, PRP LCC, I-PRF, and F-PRF injections in targeted facial areas. Ultrasound measurements provided an objective method to assess the outcomes, and statistical analysis evaluated the changes in skin density and thickness. A comprehensive literature review contextualized our findings within the broader scientific discourse on PRP and PRF applications in aesthetic medicine. Results: The protocol successfully yielded four distinct plasma fractions from a single blood draw. Ultrasound and statistical analyses demonstrated significant improvements in skin density and thickness, particularly in the lower eyelid area. These improvements were consistent across all the age groups, suggesting that the therapy’s effectiveness is independent of age. The findings also align with previous research, underscoring PRP’s and PRF’s roles in stimulating fibroblast activity, promoting collagen synthesis, and enhancing skin quality.

## 1. Introduction

Platelet-rich plasma (PRP) and platelet-rich fibrin (PRF) have been used for decades in medicine and dentistry, although the consensus on their preparation protocols, treatment regimens, or mechanisms of action remains limited. Prior studies [1,2,3,4,5] suggest that these blood-derived products exert their effects through the release of proteins and growth factors, which, when applied to the skin, promote collagen and elastin synthesis, as well as wound healing. PRP and PRF have also shown promise in treating atrophic acne scars [6], keloids [7], and melasma [8,9], and as adjunct therapies in various types of alopecia, including androgenic alopecia [10,11].

The literature on PRP/PRF for skin rejuvenation is predominantly descriptive, with few high-level studies. Only one double-blind RCT [12] and a handful of single-blind RCTs [13,14,15,16] were identified. No studies to date have reliably investigated the effects of PRP/PRF on skin based on patient age. This single-center, prospective, open-label, randomized study addresses this gap, offering valuable insights into the age-related effects of PRP/PRF on skin.

The primary goal was to develop a consistent procedure for isolating platelet-rich plasma, aiming to achieve high platelet recovery and a high concentration. Additionally, we evaluated the efficacy of autologous platelet-rich plasma (C-PRP, PRP LCC) and PRF (I-PRF, F-PRF) injections in enhancing skin density and thickness for facial aesthetic treatments. This study involved 20 participants aged 30–60 who received three treatments at 4–6 week intervals. This part of the study confirmed that C-PRP, PRP LCC, I-PRF, and F-PRF injections significantly increased the skin density and, to a lesser extent, the skin thickness in the targeted facial areas. Our findings and a detailed protocol for obtaining the four plasma fractions were recently published [1].

**Figure 1:**
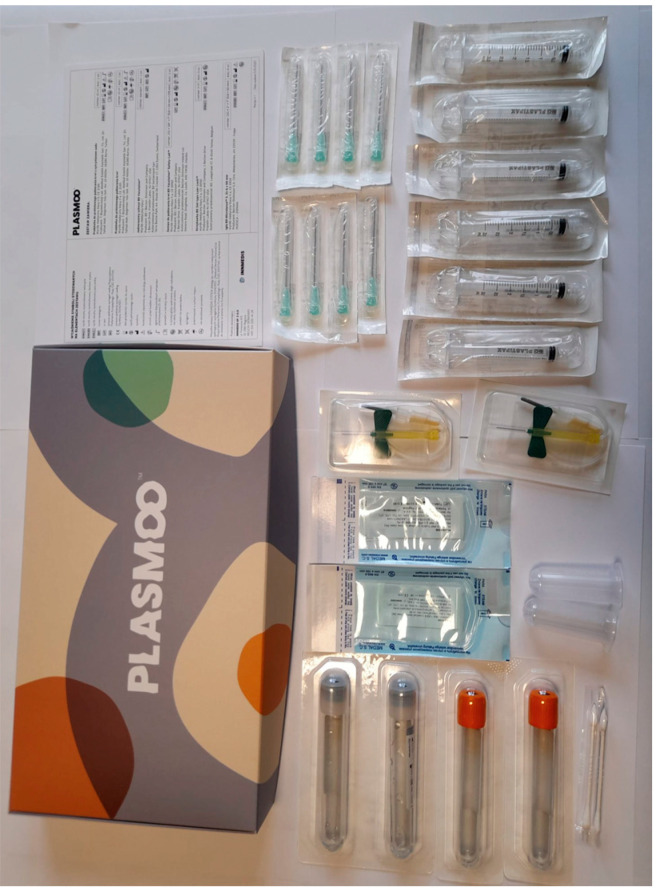
PLASMOO set (Innmedis, Kraków, Poland).

Following these results, ready-to-use PRP and PRF kits (PLASMOO, Innmedis, Poland) were developed and introduced into production (Figure 1). Statistical analysis indicated that the treatment regimen of three sessions at 4–6 week intervals effectively increased the skin density, with notable improvements in the cheek and forehead areas. The skin density and thickness in the lower eyelid also improved significantly, with high patient satisfaction, as reflected in the GAIS scores.

This study further investigated whether the effects of PRP and PRF on skin density and thickness vary by age, analyzing the results across two age groups (30–40 years, n = 9, and 40–60 years, n = 11). We consider this study a proof of concept aimed at exploring the potential efficacy of PRP and PRF injections for facial skin improvement, with a focus on age-related outcomes. Given the lack of previous literature addressing this question, our goal was to generate preliminary data as a basis for more extensive, controlled studies. Additionally, we aimed to validate high-frequency ultrasound as an objective tool for measuring skin density and thickness in aesthetic treatments.

## 2. Materials and Methods

This study consisted of two phases: a laboratory phase and a clinical phase, both detailed in a previous publication [1].

### 2.1. Laboratory Phase

**Figure 2:**
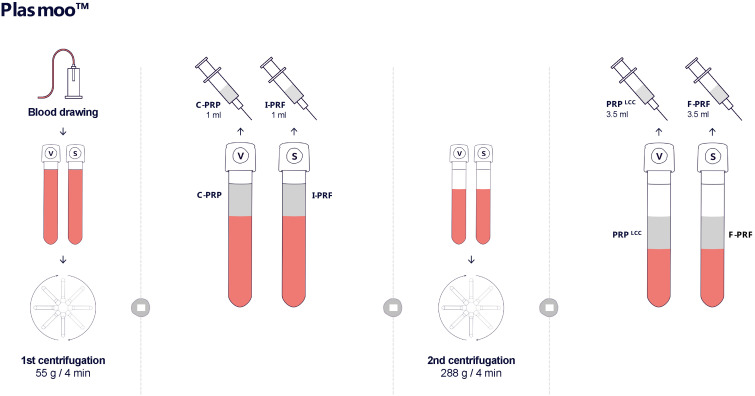
Scheme of the product preparation.

In the laboratory phase, the goal was to optimize the centrifugation parameters (time and speed) for whole blood to obtain high-quality plasma and fibrin fractions for use in aesthetic procedures. A double-centrifugation protocol was employed to produce four distinct plasma fractions: platelet-rich plasma (C-PRP and PRP LCC) and platelet-rich fibrin (I-PRF and F-PRF). These fractions were prepared in tubes with and without anticoagulant (T-LAB, Bursa, Turkey) from a single blood draw per participant. Figure 2 visualizes the preparation procedure.

During centrifugation, two platelet concentrations were achieved—202% in the first centrifugation and 148% in the second. The total platelet recovery was 76%, with an average of 32% after the first centrifugation and 44% after the second.

### 2.2. Clinical Phase

In the clinical phase, blood from each participant was collected into two tubes (one with anticoagulant and one without). Centrifugation was performed using a fixed-angle centrifuge (Neuation iFuge D06, eqlab, Tomaszów Mazowiecki, Poland) at 55 G for 4 min in the first spin and 288 G for 5 min in the second spin. Processed plasma preparations were injected into the forehead, lower eyelid, and cheek areas, which were disinfected (Skinsept, Ecolab, Monheim am Rhein, Germany) and anesthetized (Anesderm, Pierre Fabre Medicament, Castres-sur-l’Agout, France) before injection using the mesotherapy technique with 30 G/4 mm needles (Biotekne, Casalecchio di Reno, Italy).

### 2.3. Study Population

This study enrolled 20 female volunteers aged 30–60 years who were interested in facial skin rejuvenation. The participants were divided into two age groups: 30–40 years (n = 9) and 40–60 years (n = 11), allowing for a focused assessment of the age-related effects on the skin density and thickness changes. The treatments were provided at no cost. All the participants had Fitzpatrick skin types I–III and facial wrinkles classified as Glogau class II or higher.

### 2.4. Exclusion Criteria

The exclusion criteria included pregnancy or breastfeeding, blood or platelet disorders, recent facial surgery or semi-permanent dermal fillers within the past year, genetic conditions affecting fibroblasts or collagen production, a history of herpes simplex infection, active skin diseases or infections in the treatment area, a tendency for hypertrophic scarring or keloids, immunosuppressive disorders or ongoing immunosuppressive therapy, and a history of skin cancer. Participants who had used topical or oral tretinoin, received botulinum toxin or dermal fillers, or undergone chemical peels or laser treatments for facial rejuvenation in the previous six months (or planned to do so within the next six months) were also excluded.

### 2.5. Ultrasound Skin Examination

For the skin density assessment, the participants were randomly assigned for ultrasound measurements. High-frequency ultrasonography (DUB SkinScanner, tpm, Rellingen, Germany, with a 22 MHz probe) was used as the primary method to monitor the changes in skin tissue over time. The target areas included the lateral forehead, lower eyelid, and cheek. This imaging technique provides a resolution of 56 μm and allows visualization of the epidermis, dermis, and subcutaneous layers up to 16 mm deep. A focal depth of 8 mm was selected to capture potential changes in subcutaneous tissue.

The acoustic density values, normalized to a color scale, enabled comparative analysis of the collagen and elastin content changes within the dermis. These measurements were conducted manually on the ultrasound image, averaging values from a 2 mm² rectangular area. The skin thickness, measured in micrometers, was assessed by summing the epidermal and dermal thicknesses, calculated as the average of three independent measurements within each target area. The measurements were taken at baseline (prior to treatment) and one month after each treatment session.

### 2.6. Randomization and Identification

Although the participants met specific eligibility criteria (e.g., age, skin type, wrinkle severity), randomization of the measurement sessions was implemented to eliminate individual bias. The participants were identified by sequential numbering, and random selection was applied at each ultrasound measurement to ensure unbiased assessment by two researchers.

### 2.7. Aesthetic Assessment and Satisfaction

The aesthetic improvements were evaluated using the Global Aesthetic Improvement Scale (GAIS) one month after the final treatment. The GAIS scale ranges from 0 (worsening) to 4 (very significant improvement). The participants also rated their satisfaction with the treatment (very satisfied, satisfied, or not satisfied) and indicated whether they would recommend the procedure to others (yes or no).

## 3. Statistical Analysis

To quantify the results, descriptive statistics were calculated, including the mean (M), standard deviation (SD), and minimum (Min) and maximum (Max) values. The Shapiro–Wilk test was applied to assess the normality of the distribution of the studied variables. For normally distributed variables, parametric tests were used. The independent samples *t*-test evaluated the differences between two groups. For repeated measurements over time, an ANOVA test for repeated measures was applied, incorporating age as an additional qualitative variable. The Tukey post hoc test assessed the time-related changes within the parameters studied. A *p*-value of ≤0.05 was considered statistically significant. The statistical analyses were conducted using Statistica v.13.0 (StatSoft, Dell, Hamburg, Germany), with data collection and support for statistical processing performed in MS Excel 2010 (Microsoft, Redmond, WA, USA).

## 4. Results

In the participants over 40, the skin density in the forehead area showed statistically significant increases between the baseline measurement (0) and each subsequent measurement (I, II, III), with a *p*-value of 0.0001 in all the comparisons (Density 0 vs. Density I, Density 0 vs. Density II, Density 0 vs. Density III). For the participants under 40, an increase in the forehead skin density was also observed, although less pronounced. A statistically significant increase was noted between the baseline and the III measurement (Density 0 vs. Density III, *p* = 0.0014). A smaller yet statistically significant difference was found between the baseline and the I measurement (Density 0 vs. Density I, *p* = 0.0389).

**Figure 3:**
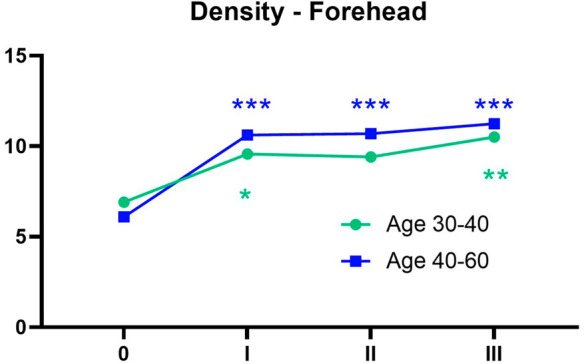
Changes in the forehead area skin density over time by age group. Green 30–40, Blue 40–60, 0 before the treatment, I one month after 1st treatment, II one month after 2nd treatment, III one month after the 3rd treatment. * *p* < 0.05, ** *p* < 0.01, *** *p* < 0.001. An asterisk next to measurement “I” indicates a statistically significant difference between measurement 1 and 0, and similarly for the other measurements. The color of the asterisks on the charts corresponds to the respective age group.

**Figure 4:**
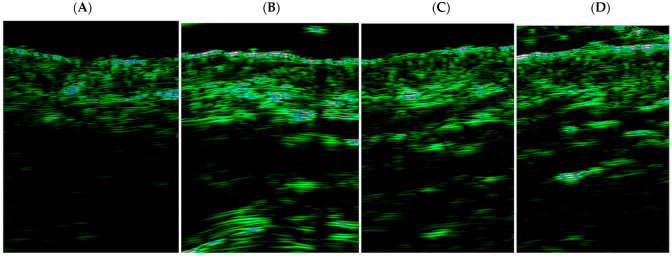
Ultrasound measurements of the forehead region—Patient #1: (**A**) before the treatment, (**B**) one month after 1st treatment, (**C**) one month after 2nd treatment, and (**D**) one month after the 3rd treatment. (The ultrasound images were acquired using a 22 MHz probe, visualizing skin structure. Green regions indicate areas of high echogenicity, representing dense structures such as the epidermis, dermis, and collagen fibers. Black regions correspond to areas of lower echogenicity, likely representing less dense connective tissue or deeper layers. Differences in green intensity reflect variations in skin thickness and density after treatment.).

The changes in the forehead skin density over time for both age groups are illustrated in Figure 3, with the corresponding ultrasound images displayed in Figure 4.

In the measurements of the lower eyelid skin density among the participants over 40 years of age, no statistically significant changes in density were observed across the measurement points (0, I, II, III). However, in the participants under 40, a statistically significant increase in density was found between the baseline measurement (0) and the second measurement (II) (*p* = 0.0351), as well as between the baseline (0) and the third measurement (III) (*p* = 0.0238).

**Figure 5:**
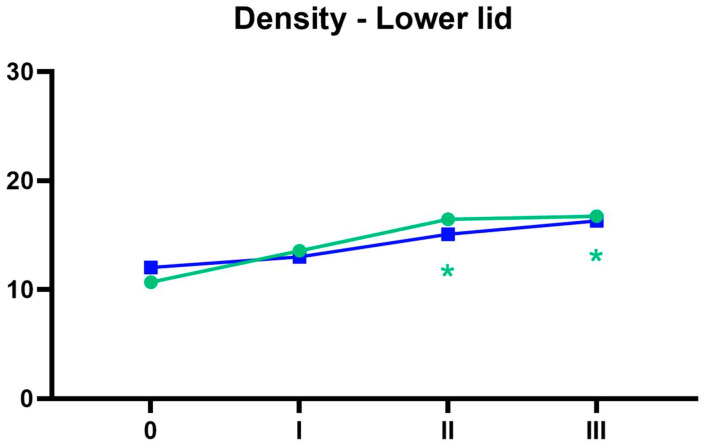
Lower eyelid skin density over time for both age groups. Green 30–40, blue 40–60, before the treatment, I one month after 1st treatment, II one month after 2nd treatment, III one month after the 3rd treatment. * *p* < 0.05. An asterisk next to measurement “I” indicates a statistically significant difference between measurement 1 and 0, and similarly for the other measurements. The color of the asterisks on the charts corresponds to the respective age group.

The changes in the lower eyelid skin density over time for both age groups are illustrated in Figure 5.

**Figure 6:**
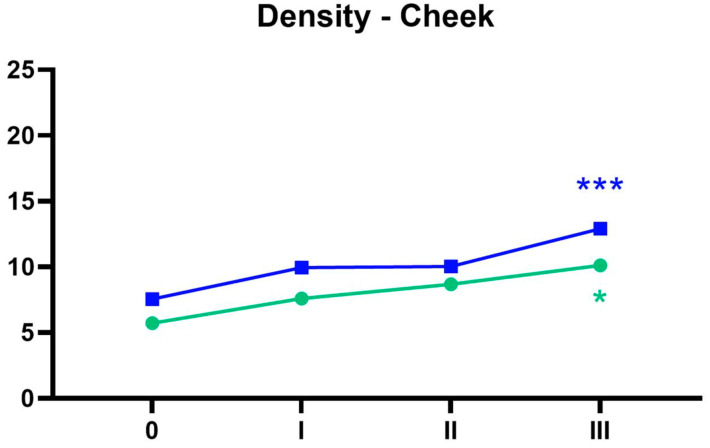
Cheek area skin density over time for both age groups. Green: 30–40. Blue: 40–60. 0—before the treatment. I—one month after the first treatment. II—one month after the second treatment. III—one month after the third treatment. * *p* < 0.05, *** *p* < 0.001. An asterisk next to measurement “I” indicates a statistically significant difference between measurement 1 and 0, and similarly for the other measurements. The color of the asterisks on the charts corresponds to the respective age group.

For the cheek area skin density in the group of individuals over 40 years old, a statistically significant increase in density was observed between the initial measurement (0) and the III measurement (*p* = 0.0003). A similar result was obtained in the group of individuals under 40 years old, where a statistically significant increase in density was also observed between the initial measurement (0) and the III measurement (*p* = 0.0116), as illustrated in Figure 6.

In the measurements of the skin thickness, statistically significant changes were observed only in the lower eyelid and cheek areas for the participants under 40 years of age. For the other parameters, such as the forehead skin thickness, no statistically significant differences were found in any of the age groups.

In the subsequent stage of analysis, a comparative evaluation between age groups was performed for each time point. This analysis used the differences between each treatment time point (I, II, III) and the baseline (0) to assess the potential variations between age groups. In the forehead, lower eyelid, and cheek areas, no statistically significant differences in the changes in skin density or thickness were identified between the participants over 40 and those under 40.

By analyzing the data by age group, we explored the potential differences in the treatment response between younger and older participants. The baseline and post-treatment measurements for each group highlighted age-related trends, although no statistically significant differences in density or thickness were found between the age groups. These results indicate that the treatment’s efficacy was not dependent on patient age.

### Survey Results

The survey results evaluating the aesthetic outcomes and patient satisfaction demonstrated high satisfaction with the applied therapy. Post-treatment, 12 participants (60%) reported being “satisfied” and 8 participants (40%) reported being “very satisfied” with the effects. These satisfaction levels aligned with a “much improved” rating on the Global Aesthetic Improvement Scale (GAIS), with an average score of 2.75.

By age group, 54.5% of patients over 40 reported being “very satisfied” compared to 45.5% who were “satisfied”. Among the younger participants, 22.2% were “very satisfied” while 77.8% were “satisfied”. The average GAIS score was 3 (much improved) for the older group and 2.5 (improved) for the younger group. The differences in satisfaction by age may reflect a more pronounced impact on older skin, where signs of aging are more noticeable, leading to higher satisfaction and perceived improvements.

**Figure 7:**
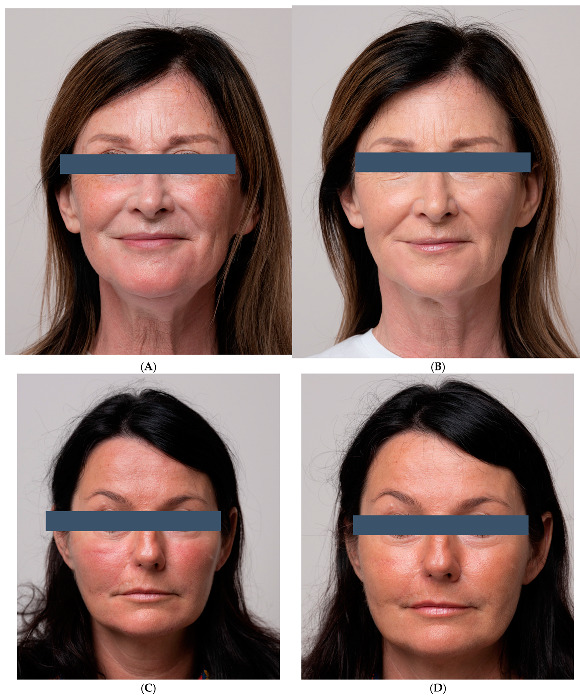
(**A**) Improvement of facial appearance, before treatment/one month after treatment completion. (**B**) Improvement of facial appearance, before treatment/one month after treatment completion. Improvement of facial appearance, (**C**) before treatment/(**D**) one month after treatment completion.

**Figure 8:**
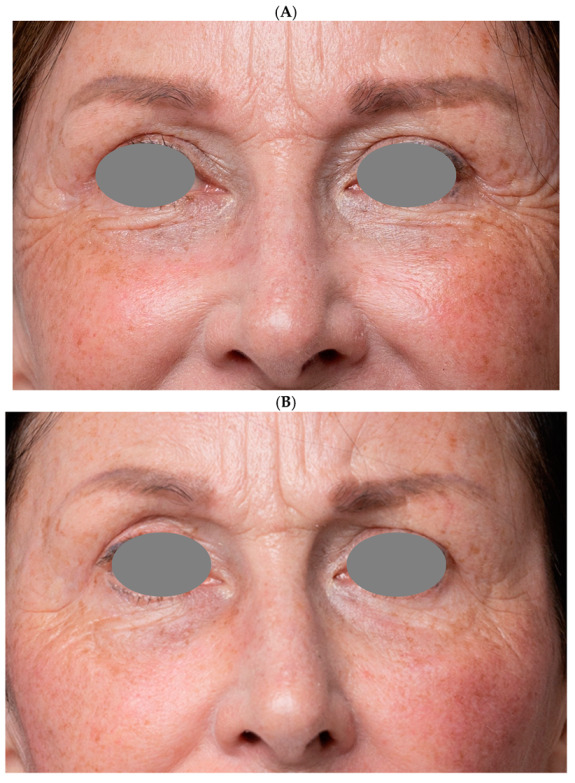
(**A**) Before treatment. (**B**) One month after treatment completion.

All the participants indicated they would recommend the treatment to others. Digital photographs taken before and after treatment show significant improvement in the skin condition (Figure 7A–D), particularly in the lower eyelid area (Figure 8A,B).

## 5. Side Effects

The adverse events reported during this study, not related to the study agent, included redness (n = 20), swelling (n = 15), and bruising (n = 11) following the injections. No adverse events were reported by any participants at the 4-month follow-up post-treatment.

## 6. Discussion

This study demonstrates that the proposed preparation protocol allows for obtaining four plasma fractions—autologous platelet-rich plasma (C-PRP, PRP LCC) and platelet-rich fibrin (I-PRF, F-PRF)—from a single blood draw. This confirms the effectiveness of these fractions in improving the skin density and thickness in specific facial areas, with the ultrasound measurement results providing objective confirmation of these outcomes [17]. Ultrasound and statistical analysis revealed significant increases in skin density in the cheek and forehead areas, as well as in both density and thickness in the lower eyelid, highlighting the effectiveness of these treatments for enhancing skin quality. These findings are consistent with other published studies in this field [18,19].

Despite various protocols for preparing blood-derived products, there remains no consensus on the optimal method, likely due to individual differences in blood parameters (e.g., hematocrit levels, erythrocytes, platelets) and diverse preparation systems. Different protocols, such as using anticoagulants or activators, variations in the centrifugation temperature, and the number and duration of the centrifugation cycles, add further variability. Numerous in vitro studies [20,21,22,23] support the effects of PRP and PRF on fibroblast activity. For instance, Cho et al. [22] demonstrated that high concentrations of PRP increase the expression of G1 cell cycle regulators, type I collagen, and MMP-1, thus accelerating wound healing. Kakudo et al. [24] found that PRP releases large amounts of PDGF-AB and TGF-beta1, favoring the proliferation of stem cells and fibroblasts derived from human adipose tissue and skin. Wang et al. [25] compared PRP with liquid PRF, showing that PRF has a greater ability to induce collagen matrix synthesis, with significantly higher mRNA levels of TGF-beta, collagen 1, and fibronectin in the PRF group.

In this study, the I-PRF injections produced the greatest improvements in the skin density and thickness in the lower eyelid area, suggesting the greater stimulatory effect of I-PRF than other plasma fractions, consistent with Wang et al. [25]. While the differences in skin thickness and density post-treatment favored younger patients in the lower eyelid area, these differences were not statistically significant, supporting I-PRF’s effectiveness across age groups. In a systematic review of 108 studies, Lei et al. [26] highlighted PRP’s role in tissue regeneration, oxidative stress reduction, and revascularization, forming the theoretical foundation for PRP’s use in facial rejuvenation.

Abuaf et al. [27] conducted a controlled placebo study that analyzed PRP’s impact on collagen production, confirming significant collagen density increases in PRP-treated areas compared to saline controls. This aligns with our findings, which suggest that PRP promotes skin density improvements via enhanced collagen synthesis. Although the current literature lacks definitive evidence regarding PRP/PRF therapy’s effects relative to age, studies like those by Vavken et al. [28] and Mori et al. [29] suggested that fibroblasts from younger patients respond more robustly to PRP treatment. Nanda et al. [30] observed that PRP might be more effective in younger patients due to the gradual decline in tissue regenerative capacity with age, while Elnehrawy et al. [31] reported the greatest wrinkle improvement from PRP in younger patients with mild to moderate nasolabial folds.

In orthopedics, mixed results concerning PRP’s age-related effectiveness have been observed, with some organizations, like the American College of Rheumatology (ACR) [32] and Osteoarthritis Research Society International (OARSI) [33], advising against PRP in knee osteoarthritis (KOA) due to insufficient data. In contrast, French experts have recommended PRP for early or moderate KOA in certain cases [34]. Age-related differences in the growth factor concentrations and inflammatory cytokine levels within PRP [35,36,37] may contribute to its varied efficacy. Some studies, such as those by Chopin et al. [38], found no significant age-related differences in the PRP response, while others argued that factors beyond age, such as immune status and metabolic health, play crucial roles in PRP’s effectiveness [39].

In the works by Trevisson et al. [40,41], the authors emphasized that age and gender should be taken into account as important modifiers of the composition of PRP. They also proposed establishing a standardized protocol that will allow for an optimized concentration of the different types of blood cells, guiding indications for prepared PRP and warning about the limitations of the different blood concentration methods, because these methods differ significantly in the quality of the obtained material, which likely affects the outcome of therapy.

Phoebe et al. [42] also recommended the appropriate selection of patients for PRP treatments based on physical and biometric data. In their study, PRP treatment led to improvements in the skin pore size, texture, wrinkle reduction, pigmented spots, and collagen density after one to three PRP sessions. Additionally, the authors concluded that combining PRP with hyaluronic acid enhanced the skin elasticity in patients with lower BMI and the skin firmness in individuals in their 50s and 60s.

The results of this study indicate that the quality and efficacy of PRP and PRF are influenced by multiple factors, with age appearing to play a minimal role. The absence of significant differences in the skin density and thickness improvements between age groups supports the notion that PRP/PRF’s effectiveness may be largely age-independent. These findings align with previous research indicating that the treatment quality and outcomes may be more influenced by individualized factors.

The influence of PRP therapy across different age groups is a subject of growing interest, not only in aesthetic medicine but also in other medical fields. Recent studies in reproductive medicine [43] have highlighted significant correlations between PRP administration and improved pregnancy outcomes, particularly in younger patients with thin endometrial linings. Research by Chang et al. demonstrated a 100% success rate in pregnancy and live births post-PRP treatment, while randomized controlled trials by Eftekar et al. confirmed increased endometrial thickness and higher implantation rates in PRP-treated patients compared to hormone replacement therapy [44,45]. Similarly, retrospective analyses by Coksuer et al. and Frantz et al. further substantiated PRP’s efficacy in reproductive applications, emphasizing its role in overcoming implantation failure [46,47].

Our study aligns with these findings, demonstrating that PRP and PRF treatments improve the skin density and, to a lesser extent, the skin thickness across different age groups. While our results suggest that the efficacy of PRP/PRF is not significantly age-dependent in facial aesthetic applications, research in reproductive medicine indicates a more pronounced benefit in younger patients. This discrepancy may be due to the differing biological mechanisms governing tissue regeneration and hormonal influences in reproductive versus dermatological applications. Future studies should explore these age-related variations further, assessing whether PRP’s regenerative capacity varies between tissue types and treatment objectives.

Recent studies in orthopedics further reinforced the relevance of age as a factor in PRP’s efficacy, with the age thresholds generally set higher than in aesthetic medicine, focusing on older patient populations. A large-scale expert consensus analysis evaluated PRP’s appropriateness for treating knee osteoarthritis (OA) across different age groups [48]. The study identified that PRP is most commonly recommended for patients aged 50–65 years, a demographic where PRP shows moderate efficacy in managing knee OA symptoms. In contrast, PRP was found to have lower efficacy in patients over 66 years old, while younger individuals (<50 years) often have higher activity expectations and may benefit from PRP as an alternative to total knee arthroplasty (TKA) [49]. This highlights the necessity of considering age when recommending PRP therapy, particularly in orthopedic applications, where degenerative changes influence treatment success. Interestingly, in cases where TKA is contraindicated due to high morbidity risks (e.g., in patients over 80 years old), PRP remains one of the few viable options, albeit with reduced effectiveness.

These findings are particularly relevant when compared to aesthetic medicine, where the majority of patients seeking PRP treatments for skin rejuvenation fall within the 30–65 age range. This aligns with our observations, as the PRP and PRF treatments in aesthetic medicine appear to be consistently effective across this broad demographic, with no significant age-related decline in efficacy. Unlike orthopedic applications, where PRP’s effectiveness diminishes in older patients due to degenerative joint changes, aesthetic PRP treatments primarily target fibroblast activity and collagen production, which seem to respond well to treatment regardless of age within this range.

This suggests that PRP is particularly well suited for aesthetic applications, where it aligns with the primary patient demographic and maintains efficacy independent of age. Future research should explore whether further refining PRP protocols could enhance the outcomes in older individuals, as seen in orthopedic applications. However, the current evidence supports the continued use of PRP in aesthetic medicine, reinforcing its broad applicability in facial rejuvenation treatments.

### 6.1. Future Perspectives

Future research should focus on conducting larger randomized controlled trials with extended follow-up durations to confirm the efficacy and durability of PRP treatments in aesthetic applications. Additionally, advancements in personalized regenerative medicine open up the possibility of tailoring PRP formulations based on individual patient profiles, such as the platelet concentration and growth factor composition. The integration of PRP with other regenerative therapies, including stem cell therapy and biomaterials, may further enhance treatment outcomes. Moreover, expanding PRP applications beyond aesthetic medicine, particularly in orthopedics, chronic wound healing, and inflammatory conditions, presents an exciting avenue for further investigation. Standardizing PRP preparation and application protocols across medical fields will be crucial to optimizing clinical outcomes and ensuring the reproducibility of results.

### 6.2. Study Limitations and Strengths

This study has several limitations, primarily due to its nature as a proof-of-concept investigation. The small sample size (n = 20), while limiting the statistical power and generalizability, aligns with early-stage research in aesthetic medicine. Our literature review on PRP/PRF treatments showed that the size of the group studied in our work is absolutely comparable to other studies in this field. For example, in a 2021 literature review on this topic [30], the authors included studies with only more than 10 patients. In the study by Lin MY et al. [5], the authors clearly stated that “Although PRP has been used clinically for skin rejuvenation for several years, the first randomized controlled clinical trial was conducted in 2018 by Alam et al.” [12], and the study itself included a group of 19 patients and assessed the effect based on patients’ subjective satisfaction and the evaluation of texture and wrinkles by two blinded dermatologists, without instrumental measurements. These studies serve as feasibility assessments, establishing preliminary findings rather than drawing definitive conclusions.

One significant limitation is the absence of a control group, such as a placebo group, which would allow for direct comparison of the treatment effects. Future research should incorporate controlled study designs to better isolate PRP/PRF’s efficacy. Additionally, further stratification of the age subcategories (e.g., 30–35, 36–40, 41–50, 51–60) could provide more granular insights into how the skin response varies with age and age-related changes. However, given the small sample size, such stratification was not feasible in this study.

The reliance on high-frequency ultrasound as the primary assessment method is a strength of this study, as it provides an objective measure of the skin density and thickness. However, incorporating histological analysis in future studies could offer direct evidence of collagen production and remodeling, which ultrasound alone may not fully capture.

While statistically significant improvements in the skin density were observed, the changes in the skin thickness were less pronounced, particularly among older participants. The clinical relevance of these findings requires further validation through patient-reported outcomes and biomechanical skin assessments.

Another potential limitation is the lack of detailed baseline participant characteristics, such as comorbidities and lifestyle factors (e.g., sun exposure, smoking, and skincare habits), which may influence skin health and confound treatment efficacy. Future studies should incorporate these variables to improve the accuracy of the findings.

Inter-individual variability in blood composition (e.g., platelet count, plasma protein levels) may also affect PRP/PRF’s efficacy. While this study aimed to standardize the preparation protocol, future research should explore personalized PRP/PRF formulations tailored to individual biological profiles.

The relatively short four-month follow-up period limits the assessment of the long-term efficacy and safety. While our follow-up duration exceeds that of many comparable studies, future research should investigate the sustainability of PRP/PRF’s benefits over extended periods and potential delayed effects.

Despite these limitations, this study’s strengths include its objective measurement approach, standardized procedures, and alignment with recognized research methodologies in the field. The use of high-frequency ultrasound enhances the replicability and provides a strong foundation for future controlled trials. Another significant strength is the practical application of the findings in aesthetic clinics. The procedures developed can be easily implemented in a wide range of clinical settings, increasing this study’s practical relevance.

As a proof-of-concept study, our findings contribute valuable preliminary data, supporting the potential efficacy of PRP and PRF treatments and laying the groundwork for further research with larger sample sizes, longer follow-up durations, and refined methodologies.

## 7. Conclusions

PRP and PRF injections demonstrate a stimulating effect on the skin density, as confirmed through ultrasound measurements. In the studied age groups, the results indicate that the treatment efficacy does not depend on the patient’s age. The standardized PLASMOO system facilitates the collection of approximately 10 mL of plasma in four distinct fractions, maximizing the use of the collected blood and providing consistent results through a standardized protocol. These findings underscore the potential of autologous plasma and fibrin formulations in aesthetic medicine, offering significant improvements in skin quality and patient satisfaction, regardless of age. Future research should further investigate the underlying mechanisms of PRP’s and PRF’s efficacy and explore refinements to enhance the protocol’s applicability in clinical practice.

## Data Availability

The data that support the findings of this study are available from the corresponding author upon reasonable request.
